# Cost-Effective Monitoring of Spruce Budworm Larvae

**DOI:** 10.3390/insects16020108

**Published:** 2025-01-22

**Authors:** Marc Rhainds, Pierre Therrien

**Affiliations:** 1 Natural Resources Canada, Canadian Forest Service-Atlantic Forestry Centre, P.O. Box 4000, Fredericton, NB E3B 5P7, Canada; 2 Ministère des Forêts, de la Faune, et des Parcs, Direction de la Protection des Forêts, Service de la Gestion des Ravageurs Forestiers, 2700 rue Einstein, Québec, QC G1P 3W8, Canada; pierre.therrien@mffp.gouv.qc.ca

**Keywords:** density-dependent dispersal, early intervention strategy, foliage protection strategy, historic valuation of metadata, Lepidopteran tree defoliators, spruce budworm risk management

## Abstract

The spruce budworm, *Choristoneura fumiferana* Clem. (Lepidoptera: Tortricidae), is the most severe defoliator of balsam fir (*Abies balsamea*) in eastern Canada and northeast U.S.A. A large budworm outbreak is currently underway in the province of Québec, with 10.5 million hectares defoliated in 2023, up 14.1% from the year before. Populations of budworms are controlled using management guidelines of Forest Protection Strategy (FPS); the approach aims at killing defoliating larvae with aerial application of microbial insecticide, with the objective to limit defoliation and prevent tree mortality. The decision to treat/not treat a given forest block is based, in part, on local density of overwintering second instars (*L2*) collected at ca. 600 sampling points each year across the entire province at a cost of CAD 350 for three branches per site; the threshold for FPS (T_FPS_) corresponds to 20 *L2*/branch. Aerial defoliation maps also guide management decisions because FPS generally target areas within or in the vicinity of defoliated forest stands. Budworm abundance rapidly declines with distance to aerial defoliation (in km), to the extent that larval density rarely attains T_FPS_ outside the core range of FPS (>15 km from defoliation). A cost-effective monitoring approach is proposed whereby forest blocks outside the core range of FPS are sampled every second year (as opposed to every year), representing a potential economy of CAD 40,000 annually.

## Simple Summary

Infestations of spruce budworm (Lepidoptera: Tortricidae) have historically been managed using aerial applications of microbial insecticide aimed to kill larvae and keep trees alive. The decision to spray is guided by aerial maps of defoliation combined with estimates of overwintering second instars per branch, using a threshold of 20 individuals. By definition, forest stands within and close to defoliated areas are prime targets of foliage protection; sites far from defoliation are less relevant to short-term management. Estimates of risk parameterized relative to distance to aerial defoliation indicated that forest blocks > 15 km away from defoliation can be sampled every second year as opposed to every year, corresponding to annual saving of ca. CAD 40,000.

## 1. Introduction

Worldwide resource management of Tortricidae pests is, by definition, very costly when considering (1) the large number of pest species; (2) the multiplicity and economic value of crop plants involved; and (3) the vast spatial extent of infestation [1,2,3,4]. In orchards of apples and other pome fruits, for example, the codling moth (*Cydia pomonella*) is present on all continents and causes severe damage (near total crop loss) because fruits infested by larvae are unmarketable [5,6]; indirect damage caused by tortricid leafrollers/defoliators also results in significant yield loss [7,8,9]. In Nearctic boreal forests, several species in the *Choristoneura* genus are major defoliators of conifers [10,11].

For both agricultural and forest tortricid pests, monitoring pest abundance is critical to forecast damage and implement management strategies. The primary objective of monitoring is to inform pest management decisions as to whether to treat or not treat a given site based on local abundance of pests; treatment is defined in generic terms and includes all control measures available for specific pest–crop associations. Management decisions are often anchored by economic thresholds, defined as the minimum pest density beyond which the benefits of treatment (increased yield/reduced tree mortality) outweigh the cost of treatment [12,13]. An exhaustive review of population monitoring in Tortricidae is beyond the scope of this study, considering the large number of pests in this taxonomic group (>687 species as of 1994) [2].

The spruce budworm, *Choristoneura fumiferana* Clemens, is the most severe pest of conifers in North America, as indicated by intensity of defoliation, correlated tree mortality, and vast geographic extent of outbreaks [14,15,16,17]. Eruptive population dynamics of spruce budworms are characterized by synchronous oscillations from endemic to epidemic phases, which tend to co-occur in space and time [18,19,20]. A large budworm outbreak is currently underway in the Canadian province of Québec (QC), characterized by high larval density and vast geographic extent of defoliation (>10.5 million ha in 2023, up 14.1% from the year before) [21,22,23].

Management of spruce budworm in QC is based on guidelines of Forest Protection Strategy (FPS), an approach aimed at killing late-feeding instars with the microbial insecticide *Bacillus thurigiensis* kurstaki (Btk) applied from the air; the ultimate objective of FPS is to protect current-year foliage of defoliated trees/prevent tree mortality in valuable forest stands [24,25,26]. Factors that influence the decision to apply Btk include the logistics of aerial treatments, ongoing schedule of harvesting, monetary value of forests to be protected, adverse effects of Btk on local community of non-target Lepidoptera, sylvicultural attributes, and proximity to waterways [27,28,29,30,31,32].

Prioritizing forest stands targeted for FPS is also guided by estimates of budworm overwintering second instars (*L2*) at a cost of CAD 350 per site (including field collection of three branches per site and laboratory processing of branches for *L2* assessment). During the last decade (2013 to 2022), abundance of *L2* was evaluated at an average of 600 sites per year, corresponding to an overall cost exceeding CAD 2 million. Cost-saving measures are proposed whereby *L2* counts are integrated with a spatial variable component (*d_i_*: nearest distance to defoliated area, in km) negatively correlated with larval abundance [33,34].

Specific research objective is to quantify risk/relevance to FPS (*Ȓ_i_*) of any forest stand on a per-year basis, using a conservative threshold *T* of 20 *L2*/branch (*T*_FPS_) [22,24,29]. For any site in year *i*, the management decision at hand is to determine whether (*Ȓ_i_* = 1) or not (*Ȓ_i_* = 0) it should be sampled in year *i +* 1. Estimates of *Ȓ_i_* are parameterized using historical data collected during a ten-year interval, focusing on probabilistic functions that larval density in offspring generations (*L2_i+_*_1_) exceeds *T*_FPS_ for any combination of larval density in parental generations (*L2_i_*) and distance to defoliation (*d_i_*).

*Ȓ_i_*~*P* (*L2_i_*_+1_ > *T*_FPS_) _(*L2i*,*di*)_ (1)

Defoliated forest stands (*d_i_* = 0) are de facto prime targets of FPS and assigned a value of *Ȓ_i_* = 1, implying that they need to be sampled every year. In contrast, sites away from defoliation with low budworm density are less relevant to FPS (*Ȓ_i_*~0) and may thus be sampled every second year. The proposed cost-saving approach infers the threshold distance to defoliation beyond which sites do not need to be sampled on a per-year basis.

## 2. Materials and Methods

### 2.1. Sources of Data

The data reported in the study are part of an ongoing survey in QC that combines densities of *L2* and defoliation maps to guide management of spruce budworm. The survey targets forest stands with balsam fir, *Abies balsamea* (L.), and spruces, *Picea* sp., as dominant or co-dominant tree species (detailed description of sampling procedures in [21,22]); only balsam fir data are included in the analyses due to the overall low number of spruce samples.

Spruce budworm aerial defoliation polygons for the years 2013 to 2023 were acquired from the Québec open data portal (https://www.donneesquebec.ca/recherche/fr/dataset/donnees-sur-les-perturbations-naturelles-insecte-tordeuse-des-bourgeons-de-lepinette (accessed on 11 June 2024)). The spatial location of each sampling point was provided by Ministère des Forêts, de la Faune et des Parcs. A custom ArcGIS.Net application (version 10.8.1) was written to calculate the distance between each sampling point and its nearest incidence to aerial defoliation polygons in the associated survey year (*d_i_*, in km). Defoliation intensity (light, moderate, or severe) is not included in analysis.

### 2.2. Statistical Analyses

Analyses were conducted with the SAS software (version 9); estimates of *L2* abundance and distance to defoliation were subjected to logarithmic transformations to reduce heterogeneity of variance. The units of observation consist of 6091 triplets collected between 2013 and 2014 and 2022 and 2023, with each triplet corresponding to a given site with estimates of *L2* in two consecutive years (*i*, *i +* 1) coupled with known distance to aerial defoliation in year *i*. Data were analyzed with glm and logistic procedures of SAS.

*L2_i_*_+1_ = *β*_0_ + *β*_1_ *L2*_i_ + *β*_2_ *d_i_* (2)

*P* (*L2_i+_*_1_ > *T*_FPS_) = e ^(*α*^_0_^+*α*^_1_*^L^*^2^*_i_*^+*α*^_2_*^d^_i_*^)^/[1 + {e^(*α*^_0_^+*α*^_1_*^L^*^2^*_i_*^+*α*^_2_*^d^_i_*^)^}] (3)

Logistically, it is assumed more practical to prioritize sites to be sampled/treated along distance classes as opposed to larval density with presumed ‘scattered’ spatial distribution. The data on the distance to defoliation were thus divided into ten class *d_ci_* bounded with multiples of 5 km [*d_ci_* = 0 for sites in defoliated areas, N = 1449; *d_ci_* = 5 for 0 < *d_i_* < 5 km, N = 576; *d_ci_* = 15 for 5 < *d_i_* < 15 km, N = 557; d_ci_ = 25 for 15 < d*_i_* < 25 km, N = 471; d_ci_ = 40 for 25 < d*_i_* < 40 km, N = 594; d_ci_ = 55 for 40 < d*_i_* < 55 km, N = 403; d_ci_ = 75 for 55 < d*_i_* < 75 km, N = 491; d_ci_ = 100 for 75 < d*_i_* < 100 km, N = 510; d_ci_ = 140 for 100 < d*_i_* < 140 km, N = 558; d_ci_ = 200 for d*_i_* > 140 km, N = 482]. For each distance class, year-to-year variation in *L2_i_*/*P* (*L2_i+_*_1_ > *T*_FPS_) (separated analyses) was derived with glm/logistic procedures of SAS.

*L2_i_* = *θ* + *θ_i_* _(*dci*)_ (4)

*P* (*L2_i+_*_1_ > *T*_FPS_) = *e ^(ϕ+ϕ^_i_^)^*/[1 + {*e^(ϕ+ϕ^_i_*^)^}] *_(dci__)_* (5)

For any combination of year/distance class, relevance to FPS (*Ȓ_ci_*) was inferred with revised Equation (1):

*Ȓ_ci_* = *k_i_* {*P* (*L2_i+_*_1_ > *T*_FPS_) _(*dci*)_} (6)

The parameter *k* was used to standardize data for each pair of year using *P* (*L2_i+_*_1_ > *T*_FPS_) in defoliated areas as the point of reference:

*k_i_* = 1/*P* (*L2_i+_*_1_ > *T*_FPS_) _(*dci* 0)_ (7)

The rules of thumb used to visualize the complex dataset at hand are described below.

## 3. Results

### 3.1. General Trends

**Figure 1:**
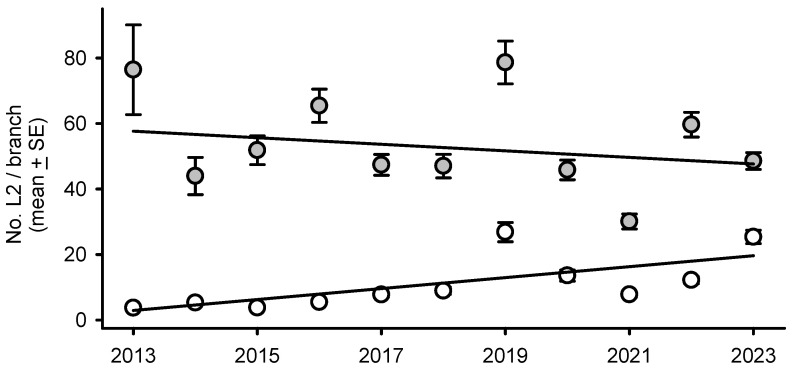
Year-to-year (x) variation in abundance of overwintering second spruce budworm per leaf of balsam fir (y = *L2_i_*) in the province of Québec. Solid lines correspond to statistically significant regressions within (grey dots: y = 2073.14 − 1.00x) and outside (white dots: y = −3360.80 + 1.67x) defoliated areas.

The density of overwintering larvae per branch in parental generations (*L2_i_*) increased over time outside defoliated forest stands at rate of 1.7 individuals per year between 2013 and 2023; within defoliated areas, in contrast, *L2_i_* values declined over time by 1.0 individuals per year on average (Figure 1).

For each pair of years between 2013 and 2014 and 2022 and 2023, future larval abundance (*L2_i+_*_1_) was positively correlated with larval density in parental generations (*L2_i_*) and inverse distance to aerial defoliation (*d_i_*) (Equation (2), Table S1). The same trends were observed with *P* (*L2_i+_*_1_ > *T*_FPS_; Equation (3)), although the effects of distance were statistically significant for only 6 of 10 pairs of years (Equation (3), Table S1).

The parameters of Equations (2) and (3) in Table S1 may, in principle, be used to derive future larval abundance for any combination of *L2_i_* and *d_i_*. Considering that *L2_i_* and *di* are both continuous variables with broad range of variation, however, year-to-year variation in effects of *L2_i_/d_i_* on *L2_i+_*_1_ are challenging to represent graphically.

### 3.2. Visual Summary of Data

**Figure 2:**
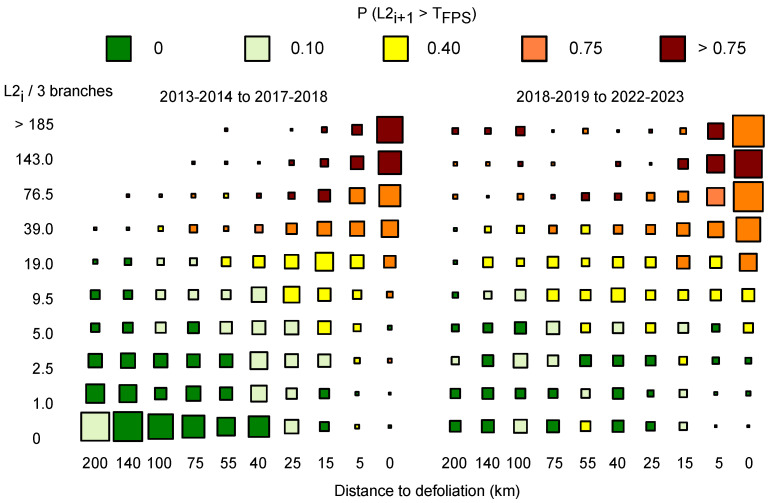
Variation in abundance of overwintering second instar spruce budworms on balsam fir (*L2_i_*/3 branches, divided into ten density classes *L2_ci_* on *y* axis) relative to distance to aerial defoliation (in km) segregated along ten distance classes *d_ci_* (upper boundary of each class on *x* axis) in the province of Québec. The threshold of the Foliage Protection Strategy (*T*_FPS_) is set at 20 *L2* per branch. For each interval, the surface and color of individual squares within 10 × 10 grids represents the number of sites with specific combinations of *L2_ci_*/*d_ci_* and the associated probability that *L2_i+_*_1_ > *T*_FPS_.

The data were divided into two intervals of equal length [early: 2013–2014 to 2017–2018; late: 2018–2019 to 2022–2023]. For each sampling interval, data were divided into ten classes along both *L2_i_* and *d_i_* dimensions, yielding *L2_ci_* and *d_ci_* (Figure 2).

(1) The early sampling interval included a vast majority (75.6%) of sites with low *L2_i_* (2730 of 3613 observations with *L2_i_* < 6.5, or *T*_FPS_/3); the proportion of ‘low density’ sites declined to 50.6% (1254 of 2478 observations) in late sampling period;
(2) The proportion of sites within 15 km to defoliation increased from 32.9% to 56.1% between sampling intervals;
(3) Consistent with statistical analyses in Table S1, *P* (*L2_i+_*_1_ > T_FPS_) increased with both *L2_ci_* and −*d_ci_*, with relatively ‘smooth’ transitions across two-dimensional classes;
(4) Transition to *L2_i+_*_1_ > *T*_FPS_ as a function of *L2_i_*/*d_i_* did not vastly differ between sampling periods, as suggested by similar color patterns in right/left plots of Figure 2.

### 3.3. Temporal Variation in Larval Density Relative to Distance to Aerial Defoliation

**Figure 3:**
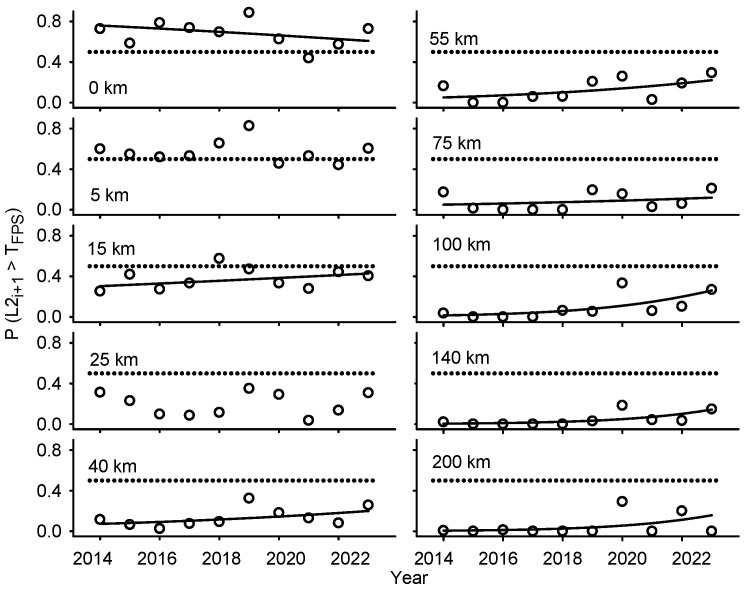
Relationships between abundance of overwintering spruce budworm second instars on balsam fir in offspring generations [y = *P* (*L2_i+_*_1_) > *T*_FPS_] as a function of year (x) for different classes of distance to defoliation in the province of Québec (upper boundary of distance class reported in each plot). Regressions parameters for each distance class, as derived with Equation (7), are summarized in Table S2. Dotted lines correspond to *P* (*L2_i+_*_1_ > *T*_FPS_) = 0.5.

Statistical analyses conducted on a per-distance-class basis (*d_ci_*) revealed similar trends in parental and offspring generations [*L2_i_* and *P* (*L2_i+_*_1_ > T_FPS_)] (Table S2, Figure 3).

(1) Monotonic decline in larval abundance with increasing distance to defoliation (parameters *θ* and *ϕ* in Equations (4) and (5));
(2) High larval abundance (consistently above or near *T*_FPS_) < 15 km from defoliation, combined with weak (inconsistent) year effect;
(3) Low larval abundance > 15 km from defoliation, generally combined with statistically significant increment over time (parameters *θ_i_* and *ϕ_i_* in Equations (4) and (5)).

**Figure 4:**
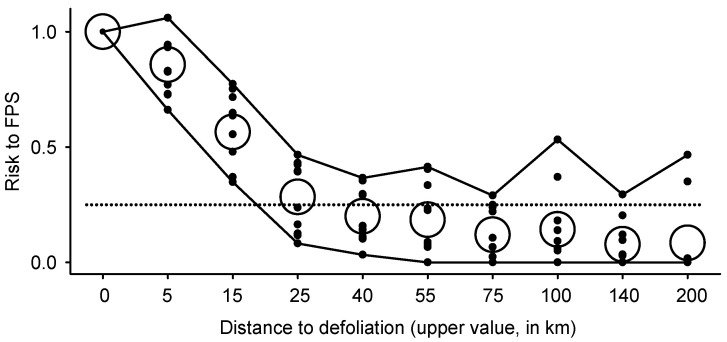
Indices of risk relative to Foliage Protection Strategy against spruce budworm (*Ȓ_ci_* as evaluated in Equation (6)) in relation with distance to defoliation (distance class *d_ci_* bounded as in Figure 1). Circles for each distance class represent average estimates for different years in the province of Québec. The bottom and upper lines represent minimal/maximal risk for any distance class. The dotted horizontal line corresponds to *Ȓ_ci_* = 0.25.

Average estimates of risk to FPS for different distance classes *d_ci_* (*Ȓ_ci_* in Equation (6)) were highest (>0.5) for sites within 15 km of defoliation and steadily declined from 0.25 to <0.10 within the spatial range *d_ci_* = 15 to 100 km (Figure 4, Table S2). The maximum value of *Ȓ* for any distance class is represented by the upper line in Figure 4.

**Figure 5:**
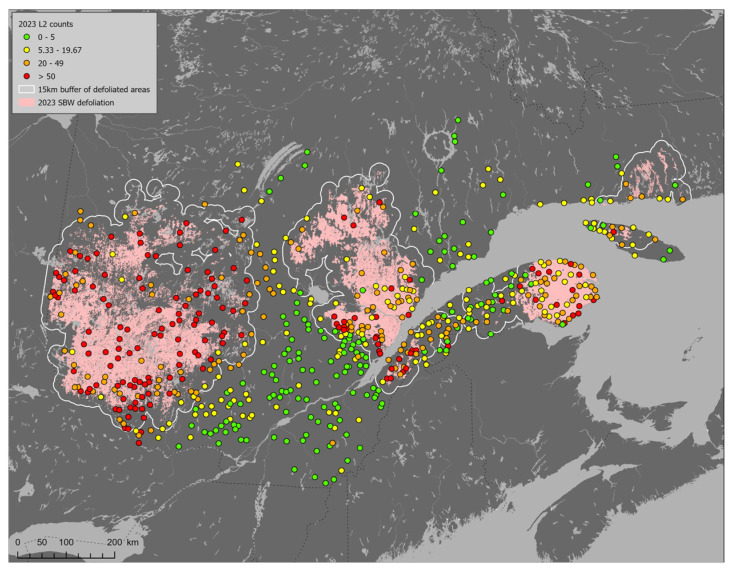
Abundance of overwintering second instar spruce budworms on balsam fir (*L2*/branch) sampled in 2023 in the province of Québec. Pink areas represent forest stands defoliated by spruce budworms based on defoliation maps. Grey lines surrounding defoliated areas represent the core range of Foliage Protection Strategy within 15 km of defoliation.

The core range of FPS was set within upper boundaries ranging between 15 to 25 km to defoliation. Geographical areas corresponding to the 15 km core range of FPS are delineated with grey contour lines in Figure 5.

## 4. Discussion

Historically, the monitoring of adult budworms has been conducted with light traps (attraction of adults of both sexes) [27,30,35,36] or pheromone-baited traps (attraction of mate-seeking males, ♂) [21,22,33,37,38]; trapping is often combined with counts of egg masses [39,40] or *L2* per host branch [41,42]. Since the early 1990s, large-scale monitoring of budworm populations in QC involved both pheromone traps and counts of *L2*, corresponding to a ca. CAD 600 cost per site. Because counts of male spruce budworms are strongly correlated with numbers of *L2* (redundant information when *L2_i_*~♂_i_ and ♂_i_~*L2_i+_*_1_) [33], pheromone-based trapping was discontinued in 2020 to reduce monitoring cost.

Still, steady growth in defoliated areas by budworms imply the ongoing budgetary constraints of monitoring. The recommendation to reduce sampling intensity from every year to every second year outside the core range of FPS (sites > 15 km away from defoliation) appears sound based on minimal risk to FPS (*Ȓ_ci_* in Equation (6), Figure 4). Implementation of this rule of thumb in 2024 would correspond to an economy of CAD 38,250. Further cost saving may be achieved by sampling out-of-range sites every *n*th year (*n* > 2), an approach that remains to be validated with field data and not promoted here.

Aerial defoliation maps correlate well with precise measurements of tree ring data [43,44] and as such provide reliable proxy of damage in multiple demographic and management contexts [14,45,46]. When coupled with distance to defoliation, *L2* abundance in parental generations provides a valuable forecasting tool to infer future larval abundance, as indicated by highly significant resolution of Equation (2) (r^2^ > 0.450 for each pair of years between 2013 and 2014 and 2022 and 2023; Table S1). Temporal increment in *L2* abundance > 15 km from defoliation may be attributed to density-dependent dispersal of egg-carrying female spruce budworms, that is, assuming that defoliated areas act as sources of migrants that amplify outbreaks over large areas [47,48,49,50].

Beyond cost issues, the historic *L2* metadata in QC are significant in terms of large- scale management of spruce budworm. For the interval 2013–2018, for example, limited defoliation in the Canadian province of New Brunswick concurred with low larval abundance rarely exceeding ‘extreme’ density > 40.5 *L2*/branch (<0.05% of 9664 sampling points), as shown in Table 1 in [51]—as also observed in QC for sampling points > 100 km from defoliation (left plots in Figure 2). Within and nearby defoliated forest stands (*d_i_* < 15 km), in contrast, *L2* density in QC commonly exceeded 40.5 *L2*/branch (58.1% of observations in Figure 2). On that basis, conclusions of population models built with an upper value of 40 *L2*/branch (~carrying capacity) must be interpreted with caution [52].

As a general conclusion, the approach developed here may be useful across a broad taxonomic range because trends of low larval density far away from crowded hosts/habitats are ubiquitous in Lepidoptera tree defoliators [53,54,55,56,57]. Factors that need to be considered include spatial scaling and distribution of trees, density- and distance functions, and major stressors (biotic or abiotic) acting on local population density.

## Data Availability

The original contributions presented in this study are included in the article and Supplementary Materials. Further inquiries can be directed to the corresponding author.

## Supplementary Materials

The following supporting information can be downloaded at https://www.mdpi.com/article/10.3390/insects16020108/s1. Table S1: Effects of larval density in parental generations (*L2i*: *β*_1_ and *α*_1_) and distance to aerial defoliation (*di*: *β*_2_ and *α*_2_, with value of zero assigned to sites within defoliated areas) on future spruce budworm larval abundance in offspring generations [*L2_i+_*_1_ or *P* (*L2_i+_*_1_ > *T*_FPS_; Equations (2) and (3), respectively]; Table S2: Year-to-year variation in abundance of overwintering second instars spruce budworms on balsam fir as a function of distance to defoliation.
