# Supplementary material for: Cost-Effective Monitoring of Spruce Budworm Larvae
Source: Insects. 2025 Jan 22;16(2):108. doi: 10.3390/insects16020108 (PMC11855817; doi:10.3390/insects16020108)
Supplement: Supplementary file 1 [file insects-16-00108-s001.zip › table S1.pdf]

Table S1. Effects of larval density in parental generations (*L2i*: $\beta_1$ and $\alpha_1$) and distance to aerial defoliation (*di*: $\beta_2$ and $\alpha_2$, with value of zero assigned to sites within defoliated areas) on future spruce budworm larval abundance in offspring generations [*L2i+1* or *P* (*L2i+1* > $T_{FPS}$; Equations 2 and 3, respectively]. Data are reported for only balsam fir and were subjected to logarithmic transformations to reduce heterogeneity of variance.

| Year i | N | $\beta_0$ | $\beta_1$ | $\beta_2$ | $r^2$ | $\alpha_0$ | $\alpha_1$ | $\alpha_2$ | Wald $\chi^2$ |
|---|---|---|---|---|---|---|---|---|---|
| 2013 | 765 | 0.770 | 0.746 *** | -0.232 *** | 0.565 *** | -3.268 | 3.584 *** | -0.488 * | 149.61 *** |
| 2014 | 724 | 0.783 | 0.670 *** | -0.302 *** | 0.662 *** | -5.154 | 4.216 *** | -0.012 | 148.39 *** |
| 2015 | 704 | 1.116 | 0.520 *** | -0.491 *** | 0.740 *** | -1.667 | 2.248 *** | -1.647 *** | 169.74 *** |
| 2016 | 777 | 0.865 | 0.560 *** | -0.268 *** | 0.716 *** | -3.948 | 3.256 *** | -0.329 | 202.62 *** |
| 2017 | 643 | 0.861 | 0.582 *** | -0.292 *** | 0.662 *** | -2.135 | 2.248 *** | -0.824 *** | 187.43 *** |
| 2018 | 488 | 1.100 | 0.623 *** | -0.192 *** | 0.691 *** | -2.688 | 4.040 *** | -0.436 * | 147.11 *** |
| 2019 | 432 | 0.333 | 0.723 *** | -0.069 * | 0.563 *** | -3.768 | 2.493 *** | 0.103 | 104.85 *** |
| 2020 | 510 | 0.976 | 0.377 *** | -0.261 *** | 0.454 *** | -1.636 | 1.053 *** | -0.795 *** | 81.56 *** |
| 2021 | 491 | 0.614 | 0.697 *** | -0.088 ** | 0.532 *** | -3.829 | 3.355 *** | -0.440 ** | 126.65 *** |
| 2022 | 557 | 0.926 | 0.552 *** | -0.089 ** | 0.516 *** | -3.298 | 3.020 *** | -0.031 | 161.27 *** |

*** : P < 0.001 **: 0.05 * : 0.15
