# Supplementary material for: Cost-Effective Monitoring of Spruce Budworm Larvae
Source: Insects. 2025 Jan 22;16(2):108. doi: 10.3390/insects16020108 (PMC11855817; doi:10.3390/insects16020108)
Supplement: Supplementary file 1 [file insects-16-00108-s001.zip › table S2.pdf]

Table S2. Year-to-year variation in abundance of overwintering second instars spruce budworms on balsam fir as a function of distance to defoliation (distance class dc_i with upper boundaries specified below). The threshold for Forest Protection Strategy (T_{PFS}) corresponds to 20 L2 / branch. Eq. 7, $L2_i$, glm procedure, parameters θ and θ_i. Eq. 8, $P\ (L2_{i+1} > T_{PFS})$, logistic procedure, parameters ϕ and ϕ_i. Regression models were simplified by using time = y – 2013, yielding models with true zero intercepts. N represents the number of sites sampled for each distance class. Data were subjected to logarithmic transformations to reduce heterogeneity of variance.

dci	*N*	θ	θ_i	ϕ	ϕ_i
0	1449	1.61	-0.015 **	1.16	-0.079 ***
5	576	1.17	0.013 *	0.38	-0.011
15	557	0.73	0.017 **	-0.83	0.060 *
25	471	0.52	0.011 *	-1.26	-0.040
40	594	0.30	0.026 ***	-2.55	0.121 **
55	403	0.27	0.037 ***	-2.92	0.184 ***
75	491	0.24	0.027 ***	-2.93	0.103 *
100	510	0.13	0.053 ***	-4.17	0.348 ***
140	558	0.09	0.046 ***	-5.32	0.392 ***
200	482	0.07	0.052 ***	-5.96	0.387 ***

*** : P < 0.001 **: 0.05 * : 0.15
